# Effects of traditional Chinese exercise therapy on pain scores, sleep quality, and anxiety-depression symptoms in fibromyalgia patients: a systematic review and meta-analysis

**DOI:** 10.1186/s12891-024-07194-7

**Published:** 2024-01-27

**Authors:** Xinzheng Wang, Hongbin Luo

**Affiliations:** Department of Physical Education, Zhejiang Chinese Medical University, Province, Zhejiang, 310053 Hangzhou China

**Keywords:** Traditional Chinese exercise, Fibromyalgia, Complementary therapy

## Abstract

**Objective:**

This study aims to assess the effectiveness of traditional Chinese exercise therapy in alleviating pain, improving sleep quality, and reducing symptoms of anxiety and depression among fibromyalgia patients.

**Methods:**

We conducted a comprehensive search across various databases, including PubMed, Cochrane Library, Embase, Web of Science, China National Knowledge, VIP database, and Wanfang, to identify randomized controlled trials (RCTs) examining the impact of Traditional Chinese Exercise (TCE) interventions on fibromyalgia. Two independent authors extracted data from the selected studies based on predefined inclusion and exclusion criteria. Meta-analyses were performed using RevMan 5.3.

**Results:**

The analysis encompassed 15 RCTs, comprising 936 participants. The meta-analysis revealed that TCE significantly surpassed the control group in reducing pain scores for fibromyalgia patients, as evidenced by improvements in FIQ [MD = -3.30, 95% CI (− 5.37, − 0.69), z = 2.53, *p* = 0.01] and VAS [MD = -1.87, 95% CI (− 2.12, − 1.61), z = 6.98, *p* < 0.00001]. Additionally, TCE demonstrated notable enhancements in sleep quality (PSQI) [MD = -2.23, 95% CI (− 2.86, − 1.61), z = 6.98, *p* < 0.0001], as well as in alleviating symptoms of anxiety and depression [MD = − 0.59, 95% CI (− 0.80, − 0.39), z = 5.63, *p* < 0.0001].

**Conclusion:**

Traditional Chinese Exercise (TCE) exhibits significant efficacy in ameliorating pain, enhancing sleep quality, and alleviating symptoms of anxiety and depression in fibromyalgia patients.

## Introduction

Fibromyalgia (FM) presents a complex and challenging chronic rheumatic disorder, characterized by a constellation of symptoms, including widespread bodily pain, sleep disturbances, fatigue, anxiety, and depression [1]. In rheumatology clinics, it ranks as the third most common diagnosis, affecting a considerable portion of the population, with prevalence rates ranging from 1.3 to 8% [2]. This enigmatic condition, often devoid of a well-defined underlying organic cause, imposes enduring physical discomfort and emotional functional limitations, profoundly compromising the quality of life for those afflicted [3]. Furthermore, FM imposes substantial direct and indirect burdens on individuals, families, and society as a whole [4]. Given the multifaceted nature of FM, a holistic and multidimensional approach to its management has been advocated. This approach extends beyond traditional pharmacotherapy and encompasses alternative interventions, such as acupuncture, electrical stimulation, massage, Specific pulsed electromagnetic fields (PEMFs) [5],cognitive therapies, and pain management strategies. Among these non-pharmacological therapies, exercise therapy has garnered significant attention and acceptance among FM patients. Research suggests that exercise can enhance daily functional capabilities, overall quality of life, and mitigate fatigue and pain [6]. In two studies, Scaturro D et al. found that the intervention regimens of Rehabilitation and Vitamin D Supplementation [7], and Exercise and Mesotherapy [8] were proven to be safe and effective in improving pain outcomes, functional recovery, and quality of life in the short term. However, in the quest to harness the therapeutic potential of exercise for FM patients, the choice of exercise modality and intensity becomes a pivotal consideration. Numerous studies have explored various exercise interventions for FM patients, including aerobic exercise, resistance training, combined aerobic and resistance training, and mind-body exercises such as Tai Chi and Qigong [9–12]. Yet, due to the often-observed reduced muscle strength and limited flexibility in FM patients, adhering to conventional exercise regimens can prove challenging, yielding less pronounced pain relief and psychological well-being improvements. Recognizing these challenges, innovative exercise strategies tailored to the specific needs and limitations of FM patients are essential for achieving more robust therapeutic outcomes. Customized exercise plans that accommodate reduced muscle strength and flexibility hold the potential to yield promising results. Thus, further investigations are warranted to determine the most effective exercise modalities and dosages to optimize pain management and enhance psychological well-being in FM patients.

In recent years, a growing body of evidence has pointed towards the efficacy of integrative mind-body exercises in alleviating FM-related symptoms [12–29]. For instance, a randomized controlled trial assessed the impact of meditative movement interventions on FM symptoms, revealing significant improvements in standardized measurements of fatigue, mood, and pain among female participants undergoing the intervention [13]. Tai Chi and Qigong have demonstrated notable effects in ameliorating severe depression [14] and improving sleep quality [15]. Tai Chi, in particular, has shown promise as an alternative approach for managing insomnia. Traditional Chinese exercise (TCE) represents a gentle and therapeutic form of low-impact aerobics and mind-body exercise derived from traditional Chinese medicine. It encompasses practices such as Tai Chi, Ba Duan Jin, Yi Jin Jing, Wu Qin Xi, and Liu Zi Jue [16–18]. TCE incorporates elements such as controlled breathing, gentle movements, mental relaxation, and meditation, contributing to cardiovascular and psychological benefits [19]. While various traditional Chinese exercise therapies like have demonstrated certain efficacy in preventing and treating chronic conditions like FM, the existing body of research consists primarily of cross-sectional or case reports, yielding inconsistent outcomes. Moreover, many investigations solely compare individual exercise therapies against no intervention, lacking a comprehensive evaluation through randomized controlled trials (RCTs) to assess the effectiveness of TCE interventions in FM patients. Therefore, this study takes a significant step towards filling this knowledge gap by focusing on six comm only used traditional fitness activities in clinical practice for FM intervention, employing RCTs as the research framework. Its primary objective is to consolidate, evaluate, and conduct a meta-analysis of existing RCTs involving TCE interventions for FM patients. By doing so, it aims to explore the evidence of efficacy for TCE interventions in the context of FM and provide valuable insights for their incorporation into health management practices.

This study adheres to the rigorous methodology outlined in the Preferred Reporting Items for Systematic Reviews and Meta-Analyses (PRISMA) guidelines [20]. Furthermore, it follows the recommendations of the PRISMA protocol and is registered in the PROSPERO database with the registration number CRD42023454469.

## Materials and methods

### Inclusion and exclusion criteria

By consensus (between X.W. and H.L.), studies meeting the following inclusion criteria and exclusion Criteria were included (Table 1), The literature was systematically screened in a sequential order.

**Table 1:** Inclusion and exclusion criteria

**Inclusion criteria:**	
(1) Participants be diagnosed with FM through hospital assessments, and they should meet the 2016 revised American College of Rheumatology (ACR) criteria for FM [1].	
(2) randomized controlled trials (RCTs)	
(3) include primary or secondary outcome measures relevant to FM patients	
(4) The experimental group’s intervention should involve traditional Chinese fitness exercises, while the control group should receive standard exercise or routine care.	
**Exclusion criteria:**	
(1) Studies that are not randomized controlled trials;	
(2) Systematic reviews and review articles;	
(3) Studies with incomplete outcome measures;	
(4) Studies with inconsistent intervention or control measures;	
(5) Conference papers.	

### Outcome measures

The primary outcome measures included Fibromyalgia Impact Questionnaire (FIQ),Visual Analogue Scale (VAS), Pittsburgh Sleep Quality Index (PSQI), Beck Depression Inventory (BDI), Hamilton Depression Scale (HAMD), State-Trait Anxiety Inventory (STAI), Hospital Anxiety and Depression Scale (HADS), Center for Epidemiological Survey, Depression Scale (CES-D). Secondary outcome measures was Widespread Pain Index (WPI).

### Literature search strategy

We conducted a comprehensive literature search by accessing multiple databases, including the Chinese National Knowledge Infrastructure (CNKI), Wanfang, VIP, PubMed, Cochrane Library, Web of Science, and Embase up to 1 October 2023. Additionally, we manually reviewed the references cited in the included studies, both in Chinese and English.

The search terms in English included “Fibromyalgia,” “Fibromyalgia syndrome,” “Chronic Fatigue Syndrome,” “Taijiquan,” “Baduanjin,” “Yijinjing,” “Wuqinxi,” “Liuzijue,” “Qigong,” “Qi Gong,” “Tai Chi,” “randomized controlled trial,” “randomized,” and “RCT.” Variants of US and British English were assumed to be encompassed within the search algorithms. For example, the search strategy employed for PubMed is presented in Table 2.

**Table 2:** Search strategy for PubMed

Search Query	
#1 Fibromyalgia [Mesh] #2 Fibromyalgia syndrome [Title/Abstract] OR (fibromyalgia syndrom [Title/Abstract] OR Fatigue Syndrome, Chronic [Title/Abstract] #3 #1 OR #2 #4 Traditional Chinese exercises [Mesh] #5 Taijiquan [Title/Abstract] OR Baduanjin [Title/Abstract] OR Yijinjing [Title/Abstract] OR Wuqinxi [Title/Abstract] OR Liuzijue [Title/Abstract] OR Qigong [Title/Abstract] OR Qi Gong [Title/Abstract] OR Ch’i Kung [Title/Abstract] OR Tai Chi Chuan [Title/Abstract] OR Tai-ji [Title/Abstract] OR Tai Chi [Title/Abstract] OR Taiji [Title/Abstract] OR Ji Quan, Tai [Title/Abstract] OR Chi, Tai [Title/Abstract] #6 #4 OR #5 #7 Randomized controlled trial [Publication Type] OR randomized [Title/Abstract] OR RCT [Title/Abstract] #8 #3 AND #6 AND #7	

### Literature selection and data extraction

Two review authors (X.W. and H.L.) independently extracted data from each included trial and resolved any disagreement through discussion. Data extraction encompassed the following aspects: (1) Year, country, and authors; (2) Baseline characteristics of the study subjects; (3) Research methodology and intervention measures; (4) Details of intervention duration, intensity, frequency, and related specifics; (5) Information regarding the control group; (6) Outcome indicators; (7) Consideration of potential biases.

### Assessment of literature quality

**Fig. 1:**
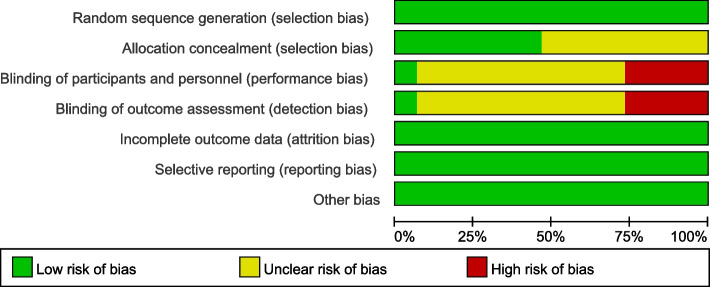
Risk of bias graph

**Fig. 2:**
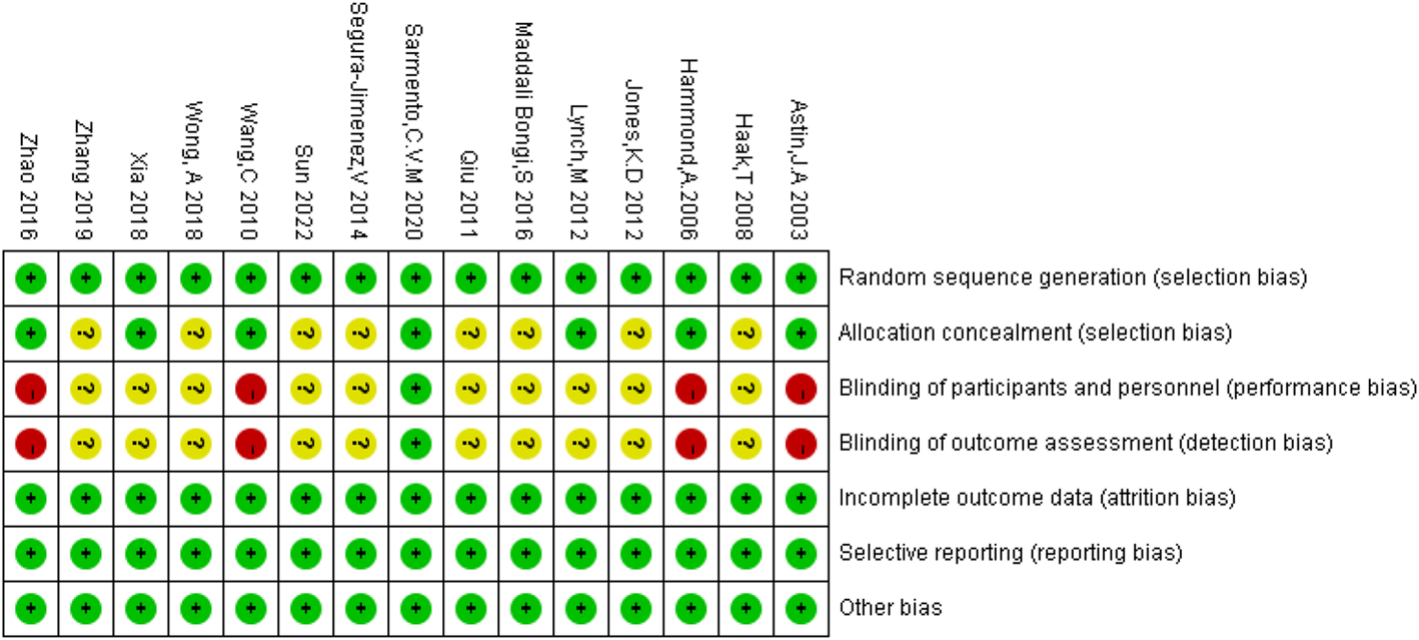
Risk of bias summary

Risk of bias assessment for each included trial was made independently by two authors (X.W. and H.L.) following the recommendations of the Cochrane Collaboration. The two authors had been previously trained in study design, research and study quality assessment etc. for qualification to perform risk of bias assessment. All 15 selected articles employed random allocation, with 10 of them explicitly detailing the randomization procedure. Figure 1 illustrates the proportional distribution of assessment criteria. Complete outcome indicators were present in all articles, denoted by a “+”, while their absence resulted in a “-” notation. “?” is unclear risk of bia (Fig. 2).

### Statistical processing

Statistical processing was performed through meta-analysis using Rev. Man 5.3 software. The quality of the literature and risks of bias were evaluated using the Cochrane tool for risk of bias assessment. The outcome indicators in the research were all continuous variables, for which we calculated weighted mean differences (WMDs) and represented the effect size using the 95% confidence interval (CI).

In terms of the heterogeneity testing of research results, we employed a fixed-effects model for analysis if I2 < 50% and *p* > 0.1. We adopted the random-effects model if I2 ≥ 50% and *p* ≤ 0.1. Any sources of heterogeneity identified in the studies were further examined through sensitivity analysis or subgroup analysis to ensure the accuracy of the research.

## Results

### Literature search results

**Fig. 3:**
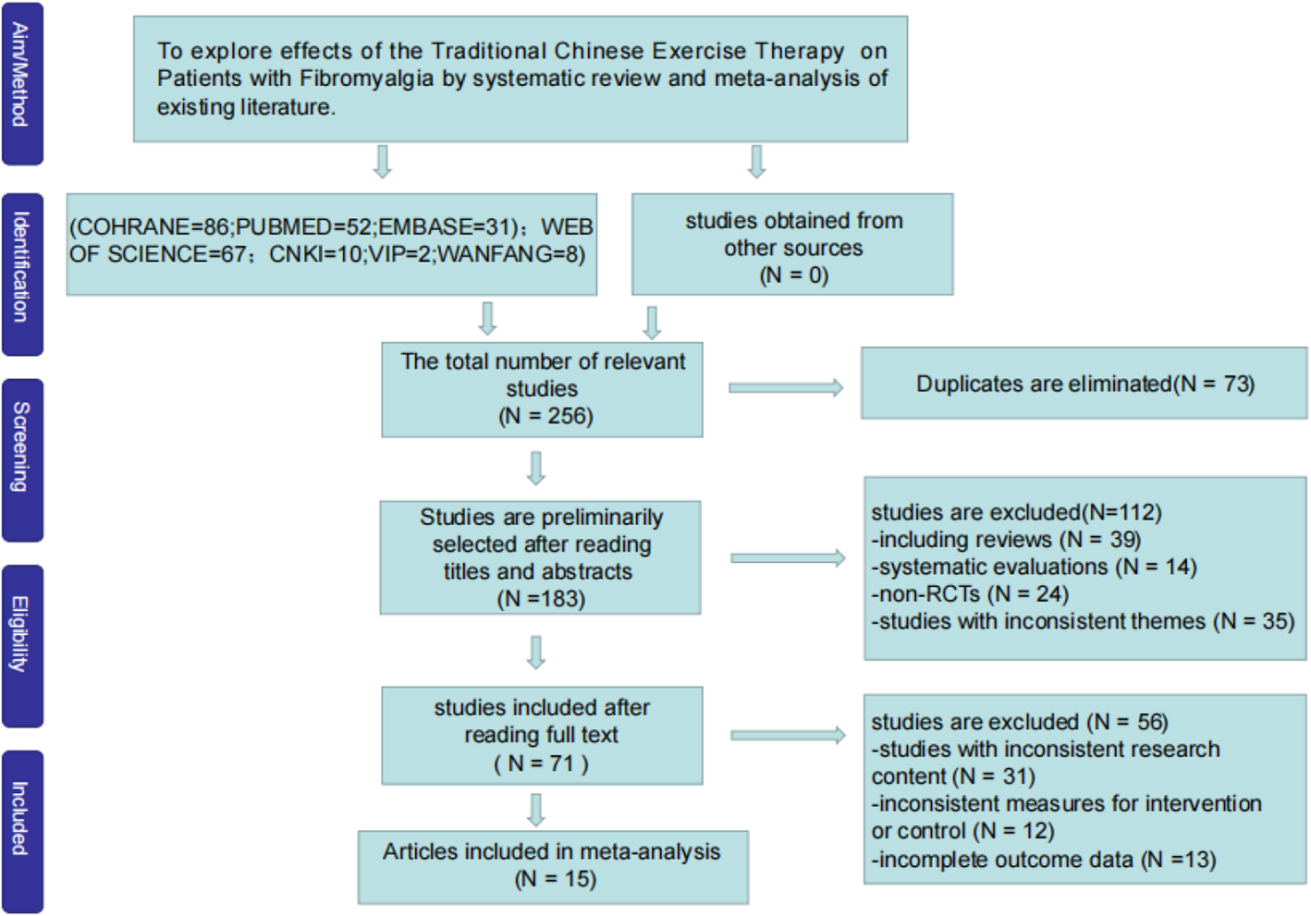
Literature selection flow diagram

A total of 256 articles were initially identified through database searches. After removing 73 duplicate articles using EndNote software, an additional 53 articles, including reviews, systematic reviews, comments, and animal studies, were excluded. Following abstract screening, 115 articles were further excluded due to incomplete data, mismatched research content, or inconsistent intervention measures. Ultimately, 15 articles were deemed suitable for inclusion in the study. The detailed process of literature selection is presented in Fig. 3.

### Characteristics and quality assessment of included studies

The 15 selected articles [15–29] were published between 2003 and 2022. The study encompassed a total of 936 participants, with 502 individuals in the experimental group and 434 individuals in the control group. The essential characteristics of the included studies are summarized in Table 3.

**Table 3:** The list of bais charateristics of inserting Meta-analysis

Literature included	Nation	Sample size (*T*/*C*)	Sex(M/F)	years since diagnosis (mean ± SD)		Age (years, mean ± SD)		Experimental group			Control group	Outcome indicator
				T	C	T	C	Content	Frequency (per week, min/times)	Duration (per week)	Content	
Zhao 2016 [21]	China	22/22	T(0/22);C(2/20)	22.27 ± 9.24	30.18 ± 20.27	48.95 ± 9.24	53.41 ± 11.01	Baduanjin	7,30	12	Regular exercise	①③④
Zhang 2019 [22]	China	21/21	Unclear	16.71 ± 7.84	15.33 ± 5.94	56.19 ± 10.88	52.90 ± 10.57	Wuqinxi	7, 30–40	12	Regular exercise	①②⑤
Xia 2018 [23]	China	21/16	T(4/17);C(3/13)	15.33 ± 5.94	16.81 ± 7.85	52.90 ± 10.57	56.19 ± 10.88	Wuqinxi	7, 30–40	12	Regular exercise	①②⑤
Sun 2022 [24]	China	73/31	T(3/28);C(11/62)	36(18–48)	21(6.5–48)	55(46–62)	53(39–58)	Baduanjin	7,30	12	Regular exercise	①②③④
Qiu 2011 [25]	China	19/20	T(3/16);C(3/17)	11.16 ± 6.01	10.15 ± 5.11	45.84 ± 8.06	44.35 ± 9.40	Yijinjing	12, 60	12	Regular exercise	①②⑤
Wong,A 2018 [26]	America	17/14	T(0/17);C(0/14)	8 ± 1	9 ± 1	51 ± 2	51 ± 2	Taiji	3, 55	12	Regular exercise	②
Wang,C 2010 [27]	America	33/33	T(5/28);C(4/29)	11.8 ± 6.9	10.0 ± 7.2	49.7 ± 11.8	50.5 ± 10.5	Taiji	7, 20	24	Regular exercise	①②④⑧
Sarmento,C.V.M 2020 [28]	America	10/10	T(0/10);C(0/10)	12.5 ± 4.7	12.6 ± 5.0	42.6 ± 10.7	56.1 ± 12.3	Qigong	14, 25	10	Regular exercise	②④⑦
Maddali Bongi,S 2016 [29]	Italy	22/22	Unclear	4.33 ± 4.03	2.36 ± 1.36	50.36 ± 13.68	54.30 ± 10.65	Taiji	2, 60	16	Regular exercise	①④⑨
Lynch,M 2012 [30]	Canada	53/47	T(3/50);C(1/46)	9.67 ± 6.87	9.62 ± 7.56	52.81 ± 8.91	52.13 ± 8.56	Qigong	7, 45–60	24	Regular exercise	①④
Jones,K.D 2012 [31]	America	51/47	T(46/5);C(43/4)	17	19	53.3	54.8	Taiji	2, 90	12	Regular exercise	①④
Hammond,A.2006 [32]	England	71/62	T(8/63);C(5/57)	2.68 ± 2.80	2.77 ± 2.95	48.36 ± 10.91	48.73 ± 10.95	Taiji	1, 45	10	Regular exercise	①
Haak,T 2008 [33]	Sweden	29/28	T(0/29);C(0/28)	15.9 ± 9.5	14.9 ± 7.5	54.0 ± 9.4	53.4 ± 8.0	Qigong	14, 40	7	Regular exercise	③⑥
Astin,J.A 2003 [34]	America	32/33	Unclear	4.89 ± 4.15	5.22 ± 7.31	40	40	Qigong	1150	8	Regular exercise	①
Segura-Jimenez,V 2014 [35]	Spain	28/28	Unclear	Unclear	Unclear	52 ± 6.6	51.7 ± 6.4	Taiji	3, 60	24	Regular exercise	②

Outcome Measures: *FIQ* Fibromyalgia Impact Questionnaire, *VAS* visual analogue scale, *BDI* Beck Depression Inventory, *PSQI* Pittsburgh Sleep Quality Index, *HAMD* Hamilton Depression Scale, *STAI* State-trait anxiety inventory, *HADS* Hospital Anxiety and Depression Scale, *CES-D* Center for Epidemiological Survey,Depression Scale, The control group received routine exercise and care (regular diet or medication management, daily walking or no exercise), *WPI* Widespread Pain Index

### Meta-analysis results

#### Effectiveness of TCE in improving pain symptoms in FM patients

**Fig. 4:**
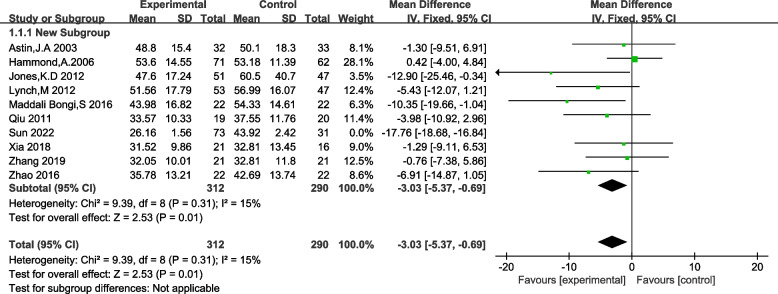
Forset plot of FIQ effect size

**Fig. 5:**
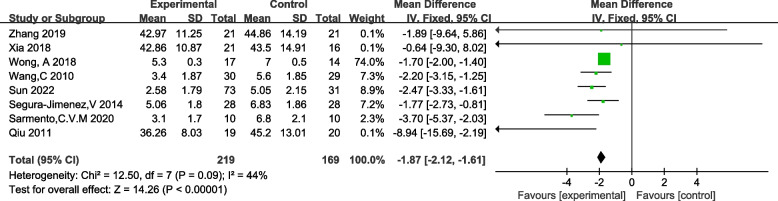
Forset plot of VAS effect size

Ten studies employing the Fibromyalgia Impact Questionnaire (FIQ) as an outcome measure, and eight studies utilizing the Visual Analogue Scale (VAS), were included in the analysis of pain scores among FM patients. Heterogeneity tests revealed no significant heterogeneity among the studies utilizing VAS. However, for studies employing FIQ, significant heterogeneity was observed with I^2^ = 94% (> 50%) and *p* < 0.01, indicating substantial variability among the selected literature. To address this heterogeneity, a sensitivity analysis was performed, revealing that the study by Sun (2022) exerted a substantial impact on the overall heterogeneity. After excluding this study, a subsequent heterogeneity test yielded I^2^ = 15%, *p* = 0.03. Consequently, a fixed-effects analysis was employed for both outcome measures. The results demonstrated a significant reduction in pain symptoms among FM patients in the traditional Chinese exercise therapy intervention group, as evidenced by the following outcomes: FIQ: [MD = -3.30, 95% CI (− 5.37, − 0.69), z = 2.53, *p* = 0.01]; VAS: [MD = -1.87, 95% CI (− 2.12, − 1.61), z = 6.98, *p* < 0.00001]. Refer to Figs. 4 and 5 for detailed information.

#### Effectiveness of TCE in improving sleep quality in FM patients

**Fig. 6:**
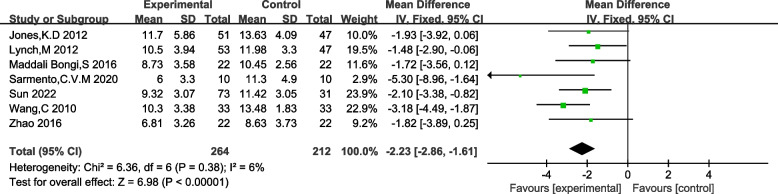
Forest plot of PSQI effect size

Seven studies utilizing the Pittsburgh Sleep Quality Index (PSQI) score as an outcome measure exhibited no heterogeneity (I^2^ = 6%, *p* = 0.38), warranting the use of a fixed-effects analysis. A lower PSQI score signifies improved sleep quality, and consistently, the intervention group demonstrated lower PSQI scores compared to the control group. The results revealed a statistically significant difference in PSQI scores between the traditional Chinese exercise therapy intervention group and the control group (MD = -2.23, 95% CI (− 2.86, − 1.61), z = 6.98, *p* < 0.0001). Please refer to Fig. 6 for detailed information.

#### Effectiveness of TCE in alleviating anxiety and depression symptoms in FM patients

**Fig. 7:**
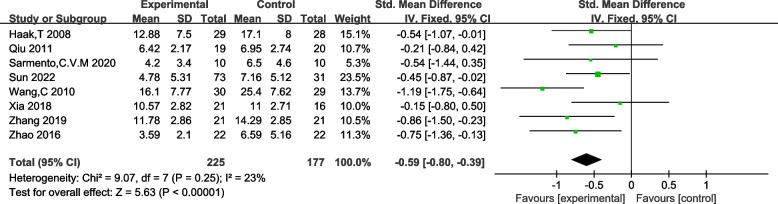
Forest plot of Anxiety and Depression Symptoms effect size

Among the eight studies included in the assessment of anxiety and depression scores in FM patients, no significant heterogeneity was observed (I^2^ = 23%, *p* = 0.25). A fixed-effects analysis was applied, revealing a statistically significant difference in the improvement of anxiety and depression symptoms among FM patients between the intervention group and the control group [MD = -0.59, 95% CI (− 0.80, − 0.39), z = 5.63, *p* < 0.0001]. Please refer to Fig. 7 for a detailed visual representation.

### Publication Bias analysis

**Fig. 8:**
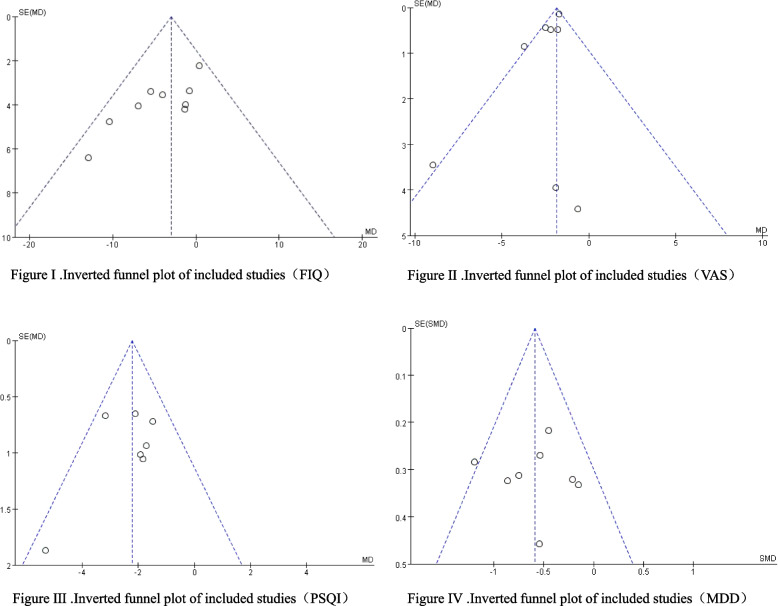
Forest plot of related indicators effect size

A funnel plot was generated to assess the potential presence of publication bias among the studies included in this meta-analysis. The assessment incorporated relevant outcome measures, including FIQ, VAS, PSQI, and anxiety and depression-related assessments. The inverted funnel plot displayed a relatively balanced distribution of positive outcomes in the published results of the included literature. Both the funnel plot and regression analysis indicated no conspicuous asymmetry, suggesting the absence of significant publication bias. These findings reinforce the robustness of the results obtained in this study. Refer to Fig. 8 for a comprehensive visualization.

## Discussion

This study set out to investigate the effectiveness of traditional Chinese exercise therapy in treating fibromyalgia (FM) patients. We included 15 randomized controlled trials (RCTs) with a total of 936 participants, the majority of whom were of advanced age. The intervention involved traditional Chinese exercise therapy, while the control groups received conventional exercise or standard care. The exclusion of confounding interventions bolstered the reliability of our findings. We assessed primary outcomes, including pain, sleep quality, and anxiety-depression status, using five distinct measurement scales: BDI, HAMD, STAI, HADS, and CES-D. A standardized mean difference (SMD) approach was employed for the meta-analysis. The results unequivocally demonstrated that traditional Chinese exercise therapy significantly reduced pain scores, improved sleep quality, and alleviated anxiety-depression symptoms in FM patients, compared to conventional exercise or standard care. A notable difference was observed between the TCE group and the control group.

Fibromyalgia (FM) represents a significant public health challenge worldwide, placing substantial strain on healthcare systems and inflicting ongoing physical and psychological distress on patients. While pharmacotherapy has historically been the primary treatment for FM, clinical trials have yet to establish a definitive therapeutic approach. The limitations of drug treatments, including dosage constraints, side effects, and incomplete effectiveness, have resulted in the limited efficacy of pharmaceutical interventions [36]. Consequently, patients seek alternative therapies such as vibroacoustic therapy, rhythmic sensory stimulation, thermotherapy, laser therapy, and exercise. Scaturro D et al. [37] discovered that the combined treatment of exercises and laser significantly improved pain perception, fatigue, and overall quality of life in patients diagnosed with fibromyalgia (FM). Exercise therapy, as a pivotal component, plays a crucial role in chronic disease management. Given the presence of symptoms like pain, fatigue, sleep disturbances, anxiety, and depression in FM patients, low-intensity and low-impact exercises are more suitable as alternative therapies. TCE blends physical activity with elements of meditation and serves as an intervention that comprehensively exercises patients’ strength, cardiovascular function, balance, and flexibility.

An accumulating body of evidence supports the pivotal role of Traditional Chinese Exercise (TCE) in mitigating pain in FM patients. One trial, involving 29 participants over 7 weeks, investigated the effects of Qigong on FM patients [33], while another study, comprising 60 participants over 24 weeks, examined the impact of Tai Chi [27]. Both studies demonstrated significant effects of these interventions on FM patients’ Fibromyalgia Impact Questionnaire (FIQ) scores and overall physical and mental well-being. Hammond et al. [33] noted a decrease in FIQ scores following a 10-week Tai Chi intervention for FM patients, indicating improvement, although substantial improvements necessitate ongoing exercise interventions. In this study, we assessed the effects of traditional exercise therapy on FM patients’ pain symptoms using two measurement dimensions: the FIQ and the Visual Analog Scale (VAS). The results revealed that traditional exercise therapy effectively improved pain symptoms in FM patients (FIQ: [MD = -3.30, 95% CI (− 5.37, − 0.69), z = 2.53, *p* = 0.01]; VAS: [MD = -1.87, 95% CI (− 2.12, − 1.61), z = 6.98, *p* < 0.00001]), consistent with the findings of the aforementioned studies. However, a systematic review of Qigong and fibromyalgia syndrome [38] found that the quality of evidence for short-term pain and sleep quality improvement with Qigong exercise was relatively low compared to conventional care interventions, which may be related to the control of the intervention process in the included studies. Among the 15 studies included in our research, the duration and intensity of exercise interventions were inconsistent. Therefore, subgroup analysis of exercise duration was not performed, indicating a need for further inclusion of high-quality randomized controlled trials for evaluation. Overall, regular and sustained traditional exercise has a pronounced effect on improving pain symptoms in FM patients.

A comprehensive study of fibromyalgia (FM) patients in China [39] identified parallels with FM patients globally, underscoring sleep quality deterioration as a core symptom. This cluster, including pain, depression, and fatigue, is closely linked to psychological well-being, emphasizing the need for interventions targeting sleep and mental health. Scholarly work by Gooneratne N [40] demonstrated a significant association between inadequate sleep in the elderly, compromised health status, and an increased prevalence of chronic diseases. Fatigue and sleep inadequacy, commonly experienced by FM patients, are often exacerbated by factors such as pain and anxiety. Roizenblatt et al. [41] discovered inferior sleep quality in FM patients, highlighting a correlation with escalated pain symptoms. An extensive survey of American FM patients [42] identified morning stiffness, fatigue, pain, attention deficits, and memory issues as particularly severe symptoms. The mechanism underlying sleep quality improvement in FM patients through TCE remains unclear, although positive effects on sleep patterns and latency reduction have been noted. Chronic pain in fibromyalgia, considered to originate from central nervous system (CNS) dysregulation, frequently coexists with sleep disturbances such as insomnia [43]. Nguyen MH et al. [44] observed significant improvement in sleep quality in a Tai Chi group among elderly Vietnamese community members. Additionally, older adults with moderate sleep problems showed enhanced sleep quality through 24 weeks of Tai Chi [45], suggesting that Tai Chi may be considered a non-pharmacological approach to addressing sleep issues in older adults with sleep disturbances. This study indicates a significant effect of traditional exercise therapy in improving sleep quality among FM patients (MD = -2.23, 95% CI [− 2.86, − 1.61], z = 6.98, *p* < 0.0001). Deterioration in sleep quality has adverse effects on physical health; therefore, implementing interventions to address sleep disturbances in FM patients can effectively alleviate FM-related symptoms. Moreover, the gentle movements, mindful meditation, and synchronized breathing of Tai Chi allow participants to engage in exercise in a comfortable and ideal state, promoting physical exertion, facilitating metabolic processes, and ultimately enhancing sleep and improving sleep quality.

Inadequate sleep, affecting approximately 10 to 50% of the population [46], is recognized to provoke adverse emotions like depression and anxiety. Poor sleep quality has been linked to inner turmoil in adults, leading to heightened susceptibility to negative emotions. The existing literature predominantly supports a bidirectional relationship between sleep and depression. A meta-analysis by Rodríguez-Almagro D incorporating 474 randomized controlled trials [47] demonstrated that appropriate doses of exercise have a moderate effect in reducing anxiety. An Asian study focusing on elderly individuals [48] revealed significant relationships between depression, anxiety scores, and sleep disorders, underscoring the complex interconnections between these elements. Sarmento CVM [49] and Cheng CA [50] et al., showed marked improvements in anxiety and depression symptoms within the taiji intervention group in FM patients. Mindfulness practices, including Tai Chi, have been shown to be effective as standalone or adjunct therapies in reducing anxiety, depression, and sleep disorders. In alignment with this research, our study indicates that Tai Chi exercise significantly improves anxiety and depression status in fibromyalgia patients (MD = -0.59, 95% CI (− 0.80, − 0.39), z = 5.63, *p* < 0.0001). When compared to other forms of aerobic exercise (running, swimming, or cycling), traditional exercise therapies like Tai Chi and Eight Brocades emphasize the integration of body and mind, achieving profound relaxation at both physical and psychological levels, thereby alleviating stress and promoting emotional stability. This intervention contributes significantly to enhancing emotional well-being, and Tai Chi exercise is a low-cost, easily implementable treatment strategy that can be seamlessly conducted within communities, demonstrating a high level of patient acceptance.

In summary, this study demonstrates the significant impact of traditional exercise therapy on various outcomes in patients with Fibromyalgia (FM), including pain, sleep quality, anxiety, and depression. Tai Chi exercise shows great potential as an adjunctive therapy for FM patients and holds promise for widespread application. However, it is crucial to strike a balance between the exercise requirements and contraindications for individuals with this condition. Before engaging in exercise, a comprehensive scientific assessment is essential. During exercise, continuous monitoring of the patient’s responses is crucial, and any adverse reactions should be addressed promptly by discontinuing the activity. These also put forward a higher demand for exercise and chronic disease prevention and treatment, exercise prescription intervention.

## Limitations of the study

While this study adhered to rigorous Meta-analysis methodology and obtained results through combining effect sizes, it is important to acknowledge certain limitations: (1) Conducting a double-blind trial for the exercise intervention is challenging, potentially leading participants in the TCE intervention group to have higher expectations. This may introduce a placebo effect that could influence the results; (2) The average age of the FM patients included in the study is relatively high, which may introduce bias and limit the generalizability of the intervention outcomes to the broader population of FM patients. (3) There were variations in the type, duration, intensity, and participants involved in the exercises included in this study, potentially contributing to the observed differences in heterogeneity. Therefore, in future Randomized Controlled Trials (RCTs), employing a more standardized experimental design and strictly controlling the intervention dosage in the treatment group will be crucial for improving the standardization and scientific validity of research related to exercise and chronic diseases.

## Data Availability

The datasets used and/or analysed during the current study available from the corresponding author on reasonable request.

## Abbreviations

TCE: Traditional chinese exercise
FM: Fibromyalgia
RCTs: Randomized controlled trials
FIQ: Fibromyalgia impact questionnaire
VAS: Visual analogue scale
BDI: Beck depression inventory
HAMD: Hamilton depression scale
STAI: State-trait anxiety inventory
HADS: Hospital anxiety and depression scale
CES-D: Center for epidemiological survey, depression scale
SMD: Standardized mean difference
OR: Odds ratio
